# Prediction of Emergent Heart Failure Death by Semi-Quantitative Triage Risk Stratification

**DOI:** 10.1371/journal.pone.0023065

**Published:** 2011-08-10

**Authors:** Harriette G. C. Van Spall, Clare Atzema, Michael J. Schull, Gary E. Newton, Susanna Mak, Alice Chong, Jack V. Tu, Thérèse A. Stukel, Douglas S. Lee

**Affiliations:** 1 Population Health Research Institute, Hamilton Health Science and McMaster University, Hamilton, Canada; 2 Institute for Clinical Evaluative Sciences, University of Toronto, Toronto, Canada; 3 Sunnybrook Health Sciences Centre, University of Toronto, Toronto, Canada; 4 The Dalla Lana School of Public Health, University of Toronto, Toronto, Canada; 5 Mt. Sinai Hospital, University of Toronto, Toronto, Canada; 6 Toronto General Hospital and University Health Network, University of Toronto, Toronto, Canada; University of Modena and Reggio Emilia, Italy

## Abstract

**Objectives:**

Generic triage risk assessments are widely used in the emergency department (ED), but have not been validated for prediction of short-term risk among patients with acute heart failure (HF). Our objective was to evaluate the Canadian Triage Acuity Scale (CTAS) for prediction of early death among HF patients.

**Methods:**

We included patients presenting with HF to an ED in Ontario from Apr 2003 to Mar 2007. We used the National Ambulatory Care Reporting System and vital statistics databases to examine care and outcomes.

**Results:**

Among 68,380 patients (76±12 years, 49.4% men), early mortality was stratified with death rates of 9.9%, 1.9%, 0.9%, and 0.5% at 1-day, and 17.2%, 5.9%, 3.8%, and 2.5% at 7-days, for CTAS 1, 2, 3, and 4–5, respectively. Compared to lower acuity (CTAS 4–5) patients, adjusted odds ratios (aOR) for 1-day death were 1.32 (95%CI; 0.93–1.88; p = 0.12) for CTAS 3, 2.41 (95%CI; 1.71–3.40; p<0.001) for CTAS 2, and highest for CTAS 1: 9.06 (95%CI; 6.28–13.06; p<0.001). Predictors of triage-critical (CTAS 1) status included oxygen saturation <90% (aOR 5.92, 95%CI; 3.09–11.81; p<0.001), respiratory rate >24 breaths/minute (aOR 1.96, 95%CI; 1.05–3.67; p = 0.034), and arrival by paramedic (aOR 3.52, 95%CI; 1.70–8.02; p = 0.001). While age/sex-adjusted CTAS score provided good discrimination for ED (c-statistic = 0.817) and 1-day (c-statistic = 0.724) death, mortality prediction was improved further after accounting for cardiac and non-cardiac co-morbidities (c-statistics 0.882 and 0.810, respectively; both p<0.001).

**Conclusions:**

A semi-quantitative triage acuity scale assigned at ED presentation and based largely on respiratory factors predicted emergent death among HF patients.

## Introduction

Heart failure (HF) is a major cause of cardiovascular morbidity and a leading reason for emergency department (ED) utilization, with over one million visits to the ED each year in North America [1], [2]. Not only is the mortality rate of HF high, but the direct and indirect annual costs of HF are estimated to exceed $1 billion in Canada and $37 billion in the United States; 70–80% of these costs are attributed to patients cared for in the ED or admitted to hospital [3].

Management of cardiac patients is largely predicated on the risk of adverse outcomes, but there are large knowledge gaps in prognostication in the emergent care of acute HF [4], [5]. While there have been several risk prediction models developed for assessment of hospitalized patients with acute HF [6]–[10], there is a paucity of risk stratification tools intended for use in the ED which includes those who are admitted or discharged. The Canadian Triage Acuity Scale (CTAS) was developed to allow healthcare providers in the ED to evaluate patients' acuity level and needs for timely care for a broad range of emergency conditions. Patients are routinely assigned a CTAS level by the triage nurse in the ED on the basis of a 2–5 minute assessment, which may include one or more of the following: presenting complaint, mechanism of injury, and symptom severity (**Table 1**). The numeric CTAS system also makes a proviso for empiric judgment, whereby the healthcare provider is allowed to scale up the acuity level if a patient is perceived to be more unwell than designated by the scale. Increasing CTAS scores correspond to decreasing acuity of illness: 1 = resuscitation/critical, 2 = emergent, 3 = urgent, 4 = less urgent, and 5 = non-urgent [11].

**Table 1:** CTAS classification for HF/dyspnea.

CTAS Level	Symptom Severity	Oxygenation Saturation	Time to MD assessment	Nursing reassessment
1 (Resuscitation/Critical)	Fatiguing from excessive work of breathing, cyanosis, single word speech, upper airway obstruction, imminent cardiac arrest or shock	<90%	Immediate	Continuous
2 (Emergent)	Increased work of breathing, speaking phrases or clipped sentences, significant or worsening stridor, protected airway	90–92%	Within 15 minutes	Every 15 minutes
3 (Urgent)	Dyspnea, tachypnea, shortness of breath on exertion, no obvious increased work of breathing, able to speak in sentences, stridor without obvious airway obstruction	>92%	Within 30 minutes	Every 1 hour
4 (Semi-urgent)	Less urgent with potential for deterioration or complications	>95%	Within 1 hour	Every 1 hour
5 (Non-urgent)	Chronic problem without evidence of deterioration; Investigations or interventions could be referred to other areas of hospital or health care system	>95%	Within 2 hours	Every 2 hours

The CTAS is a generic scale that is used to guide work flow in the ED, with higher-acuity ratings translating into need for higher prioritization in the patient queue, better monitoring in the treatment area, and more rapid assessment by a physician. However, it is not known whether this semi-quantitative score can be extended to predict acute outcomes among HF patients who have high acute mortality. Demonstration of a significant association of higher initial triage acuity with mortality risk may enhance the usefulness of this scale in physicians' management and discharge plans.

The objective of this study was to assess the utility of CTAS in evaluating the risk of emergent or early mortality among HF patients in the ED, where emergent death was defined as those occurring within 1 day of emergency department presentation. We also sought to determine if the predictive ability of the CTAS could extend to a longer-term 30-day mortality window. We also assessed the clinical characteristics, discharge disposition, and emergency department and follow-up care of HF patients according to the CTAS.

## Methods

### Ethics statement

Research ethics board approval was obtained from Sunnybrook Health Sciences Centre, who deemed that written informed consent was not required and waived the need for informed consent as this was an analysis of linked administrative databases.

### Study patients

We performed a retrospective cohort analysis drawn from the population of Ontario, Canada, comprised of patients presenting to one of 182 EDs with acute HF, using the National Ambulatory Care Reporting System (NACRS) database. Patients visiting the emergency department were eligible for this study if they had HF as the main ED diagnosis, based on the International Classification of Diseases, 10th edition (ICD-10-CA) coding system, code I50 [12]. Patients were included if they were ≥18 years of age, and they presented to an ED with HF from Apr 1, 2003 to Mar 31, 2007. A validation study that assessed a chart abstraction cohort (n = 3623) of ED patients discharged with a main ICD-10 diagnosis code I50 revealed that 92.7% either fulfilled Framingham criteria, were hospitalized with a HF diagnosis, or had ≥2 physician billing fee codes for HF, suggesting high predictive value for clinical heart failure [13]. In those with multiple ED visits during the study period, the first visit was defined as the index HF episode. Patients were excluded if they had invalid health card numbers, were non-residents of Ontario, had missing demographics (e.g. gender, income quintile), were >105 years of age, left the ED against medical advice or prior to being seen/treated, declined treatment, were transferred to another facility, or were transferred to an ambulatory day centre within the same facility. Transferred patients were excluded because we were interested in the association between triage assessment, processes of care in the ED, and short-term clinical outcomes, which would not be directly evaluable if transferred patients were included.

### Data sources

Emergency department data were obtained from NACRS, which contains information on all visits occurring at any ED in the province of Ontario; Hospitalization information was obtained via the Canadian Institute for Health Information Discharge Abstract Database (CIHI-DAD), and both databases were linked using the patients' unique encrypted health card number. We examined all records in the CIHI database for 5 years prior to the index ED visit in order to determine: the number of admissions for HF or myocardial infarction (MI) preceding the index ED visit, coronary revascularization procedures (e.g., coronary artery bypass graft [CABG] or percutaneous coronary intervention [PCI]), cardiac device implantation (e.g., implantable cardioverter defibrillator [ICD] or permanent pacemaker [PPM]), comorbidities, and other cardiovascular diseases. Hospitals were divided into three categories according to the Joint Policy and Planning Committee into teaching, large community, and small institutions.

### Chart Abstraction

To examine the clinical correlates of triage-critical status (CTAS 1), primary charts were abstracted from 32 hospitals by highly-trained and experienced nurse chart abstractors for information on ED presentation, including demographic factors, vital signs, past cardiac and non-cardiac medical history, and co-morbidities. Patient charts were randomly selected for abstraction if the patient visited an ED in Ontario for a primary diagnosis of HF (ICD-10 code I50) during the period from April 1, 2004 to March 31, 2007.

### Outcomes

Our primary outcomes were emergent death occurring either in the ED or within 1 day from the date of presentation. To determine if CTAS was associated with early mortality, we also examined the secondary outcomes of death within 7 and 30 days after ED presentation. Outcome events were detected by examining the Registered Persons Database (RPDB) of vital statistics, NACRS, and the CIHI-DAD.

### Analysis

We compared baseline characteristics, past medical history, pre-ED care, presentation features, use of specialist services, disposition, and outcomes of patients according to the CTAS score. We compared continuous variables using linear regression, and categorical variables using the Mantel-Haenszel χ^2^ test for trend. Kaplan-Meier survival curves were constructed stratified by CTAS score. Using multiple logistic regression, we compared the performance of a model including only age, sex, and CTAS vs. a previously-published multivariable model [14] including age, sex, cardiac and non-cardiac disease, ED length of stay, and the CTAS, by comparing areas under the receiver operating characteristic (ROC) curves using STATA (College Station, TX). We examined patient-related predictors of triage critical CTAS score (level 1) using a non-parsimonious logistic regression model constructed with the following covariates: demographics (age <70 years, male sex); vital signs (heart rate >120 beats/min, systolic blood pressure <100 mmHg, respiratory rate >24 breaths/min, oxygen saturation <90%); cardiac presentation (cardiac or respiratory arrest during transit to ED, NYHA class IV, ongoing chest pain); co-morbidities; mode of transportation (ambulance); overnight arrival to the ED (between 0000 and 0600 hours); hospital type (teaching, large community, or small community); and recent ED visit or hospital discharge (within 7 days). We constructed a parsimonious model using stepwise selection retaining only those covariates with p<0.05 in the multivariable model. We assessed model fit using the Hosmer-Lemeshow statistic, and model discrimination using the c-statistic which is equivalent to the area under the receiving operator characteristic (ROC) curve [15]. Analyses were performed using SAS version 9.1.3 (Cary, NC).

## Results

### Patient Characteristics

A total of 106,393 HF records were screened in the NACRS database for study eligibility. Of these, patient visits were excluded because they were <18 or >105 years of age (n = 175; 0.2%) or were non-residents of Ontario (n = 148; 0.1%). The remaining 106,070 ED visits for HF arose from 70,114 unique patients seeking acute care. Of these, we excluded patients who left without being assessed (n = 7; 0.01%) or against medical advice (n = 234; 0.3%), those transferred to another facility (n = 1327; 1.9%), and those without a CTAS rating (n = 166; 0.2%) leaving a final study cohort of 68,380 HF patients.

The mean age in the cohort was 76±12 years, and 49.4% of the patients were men. Among the 68,380 patients, 3.1% were assigned a triage-critical score of CTAS 1 - the highest acuity rating. Intermediate acuity scores of CTAS 2 and 3 were assigned to 40.4% and 46.8% of HF patients, respectively. CTAS scores 4 or 5 were assigned to 9.7% of all patients presenting to the ED with HF. There was a significant trend towards older age and female sex in those of higher acuity (i.e. lower CTAS levels). A greater proportion of patients with higher acuity levels had prior MI, HF, CABG, or PCI. There was no significant difference between CTAS groups in the proportion of patients with prior ICD or PPM implantation. Patients with higher acuity levels had greater prevalence of diabetes, peripheral vascular disease, and renal disease. The prevalence of other co-morbidities in the cohort is presented in **Table 2**. The prevalence of peptic ulcer disease (1.9–2.2% across CTAS groups), hemi-/paraplegia or disability (2.0–2.9% across CTAS groups), protein-calorie malnutrition (0.9% for all CTAS groups), prior pneumonia (14.3–16.6% across CTAS groups), and major psychiatric disorders (5.2–6.1% across CTAS groups) was not significantly different across CTAS categories and is not shown in **Table 2**.

**Table 2:** Patient characteristics by CTAS level.

Characteristic^1^	CTAS 1 (Highest acuity)	CTAS 2	CTAS 3	CTAS 4–5 (Lowest acuity)	p-value
N	2,136	27,614	31,998	6,632	
Age, yrs, mean(SD)	76.1 (11.5)	76.1 (11.6)	76.7 (11.6)	75.9 (11.9)	0.023
Male, n(%)	1,033 (48.4%)	13,491 (48.9%)	15,900 (49.7%)	3,356 (50.6%)	0.003
Systolic blood pressure, mean(SD)^2^	158.5 (36.3)	147.8 (30.9)	144.4 (26.9)	141.6 (25.4)	<.001
Heart rate, mean(SD)^2^	105.5 (28.6)	88.5 (24.1)	84.3 (19.8)	81.8 (17.0)	<.001
Respiratory rate, mean(SD)^2^	28.8 (8.5)	23.4 (7.0)	21.3 (5.1)	20.4 (6.4)	<.001
Prior myocardial infarction, n(%)	458 (21.4%)	5,407 (19.6%)	5,142 (16.1%)	891 (13.4%)	<.001
Prior heart failure, n(%)	670 (31.4%)	8,411 (30.5%)	9,294 (29.0%)	1,816 (27.4%)	<.001
Recent CABG, n(%)^3^	22 (1.0%)	484 (1.8%)	501 (1.6%)	72 (1.1%)	0.012
Recent PCI, n(%)^3^	48 (2.2%)	523 (1.9%)	415 (1.3%)	68 (1.0%)	<.001
Recent ICD implant, n(%)^3^	10 (0.5%)	117 (0.4%)	102 (0.3%)	23 (0.3%)	0.059
Recent pacemaker, n(%)^3^	19 (0.9%)	386 (1.4%)	426 (1.3%)	82 (1.2%)	0.819
Chronic atherosclerosis, n(%)	622 (29.1%)	8,288 (30.0%)	8,615 (26.9%)	1,706 (25.7%)	<.001
Prior cardiopulmonary arrest, n(%)	123 (5.8%)	1,126 (4.1%)	1,127 (3.5%)	217 (3.3%)	<.001
Valvular heart disease, n(%)	140 (6.6%)	2,146 (7.8%)	2,300 (7.2%)	410 (6.2%)	<.001
Peripheral vascular disease, n(%)	136 (6.4%)	1,567 (5.7%)	1,668 (5.2%)	351 (5.3%)	0.006
Cerebrovascular disease, n(%)	169 (7.9%)	2,037 (7.4%)	2,440 (7.6%)	470 (7.1%)	0.746
Diabetes, n(%)	365 (17.1%)	4,579 (16.6%)	4,933 (15.4%)	992 (15.0%)	<.001
Respiratory disease, n(%)	404 (18.9%)	4,706 (17.0%)	5,287 (16.5%)	1,150 (17.3%)	0.147
Renal disease, n(%)	287 (13.4%)	3,396 (12.3%)	3,635 (11.4%)	631 (9.5%)	<.001

1 Within previous 3 years of ED visit unless otherwise indicated.

2 Vital signs available in patients with chart-abstracted data.

3 Within previous 6 months of ED visit.

CABG = coronary artery bypass graft surgery, PCI = percutaneous coronary intervention, ICD = implantable cardioverter defibrillator.

### Pre-emergency Department Care

Less than one-third of the patients in each CTAS category (27.8% to 32.3%) received outpatient care within the week preceding their ED presentation (**Table 3**). Compared to the other acuity levels, CTAS 1 patients were less likely to have received medical care in the week (27.8%) or month (51.6%) prior to ED presentation, compared to CTAS 2 or 3 patients.

**Table 3:** Care of patients prior to their index HF presentation in the ED.

	CTAS 1 (Highest acuity)	CTAS 2	CTAS 3	CTAS 4–5 (Lowest acuity)	p-value
Total N	2,136	27,614	31,998	6,632	
Recent visit (w/in 7 days) to:					
a) Any MD, n(%)	594 (27.8%)	8,897 (32.2%)	10,335 (32.3%)	1,944 (29.3%)	0.236
b) Cardiologist, n(%)	45 (2.1%)	636 (2.3%)	638 (2.0%)	114 (1.7%)	0.002
c) Primary care, n(%)	427 (20.0%)	6,861 (24.8%)	8,011 (25.0%)	1,561 (23.5%)	0.356
d) Other specialist, n(%)	167 (7.8%)	2,226 (8.1%)	2,628 (8.2%)	425 (6.4%)	0.013
Prior visit (w/in 30 days) to:					
a) Any MD, n(%)	1,303 (61.0%)	18,717 (67.8%)	21,764 (68.0%)	4,263 (64.3%)	0.529
b) Cardiologist, n(%)	140 (6.6%)	2,300 (8.3%)	2,413 (7.5%)	337 (5.1%)	<.001
c) Primary care, n(%)	1,042 (48.8%)	15,353 (55.6%)	18,166 (56.8%)	3,607 (54.4%)	0.003
d) Other specialist, n(%)	492 (23.0%)	7,351 (26.6%)	8,471 (26.5%)	1,505 (22.7%)	0.002

### Emergency Department Care

Large community hospitals provided care to a greater proportion of patients with high acuity levels than other hospital types, while the proportion of patients within each CTAS level presenting to small community hospitals increased with decreasing illness acuity (**Table 4**). Patients in higher acuity categories more often presented with paramedic assistance. The time of arrival to the ED also varied significantly among CTAS groups; while a majority of CTAS 1 patients arrived in the early morning (between midnight and 0759 hours), most patients belonging to other acuity groups arrived during daytime hours (0800 to 1700 hours). A majority patients with HF, including those with the highest acuity scores, did not receive consultation from a cardiologist or internist and a trend in the rates of specialist consultation was present (**Table 4**). Use of specialist consultative services for patients across all acuity levels was most frequent in teaching hospitals, and least frequent in small community hospitals.

**Table 4:** Acute and post-emergency care of HF patients presenting to the ED.

	CTAS 1 (Highest acuity)	CTAS 2	CTAS 3	CTAS 4–5 (Lowest acuity)	p-value
Total N	2,136	27,614	31,998	6,632	
***Hospital Type***					
Teaching, n(%)	382 (17.9%)	5,050 (18.3%)	6,319 (19.7%)	802 (12.1%)	<.001
Large Community, n(%)	1,704 (79.8%)	21,824 (79.0%)	23,432 (73.2%)	3,855 (58.1%)	
Small Community, n(%)	50 (2.3%)	740 (2.7%)	2,247 (7.0%)	1,975 (29.8%)	
***Mode of arrival:***					
Paramedic, n(%)	1,741 (81.5%)	14,150 (51.2%)	12,781 (39.9%)	1,508 (22.7%)	<.001
***Time of initial ED arrival:***					
Day [0800 to 1700], n(%)	701 (32.8%)	13,962 (50.6%)	19,159 (59.9%)	4,568 (68.9%)	<.001
Evening [1701 to 2400], n(%)	578 (27.1%)	7,005 (25.4%)	7,670 (24.0%)	1,356 (20.4%)	
Early AM [0001 to 0759], n(%)	857 (40.1%)	6,647 (24.1%)	5,169 (16.2%)	708 (10.7%)	
***Seen by consultant in ED:***					
Cardiologist, n(%)	241 (11.3%)	1,748 (6.3%)	1,531 (4.8%)	117 (1.8%)	<.001
Internist, n(%)	745 (34.9%)	7,536 (27.3%)	7,724 (24.1%)	1,010 (15.2%)	<.001
Other specialist, n(%)	229 (10.7%)	2,556 (9.3%)	2,139 (6.7%)	164 (2.5%)	<.001
***Disposition (all patients):***					
Admitted to ICU/CCU, n(%)	680 (31.8%)	3,695 (13.4%)	2,289 (7.2%)	295 (4.4%)	<.001
Admitted to ward, n(%)	1,234 (57.8%)	18,233 (66.0%)	18,455 (57.7%)	2,506 (37.8%)	
Discharged home from ED, n(%)	91 (4.3%)	5,578 (20.2%)	11,218 (35.1%)	3,819 (57.6%)	

### Disposition from the ED

Only 31.8% of patients assigned to CTAS 1 were admitted to an intensive care or coronary care unit. Among patients assigned to CTAS scores 2, 3, and 4–5, 13.4%, 7.2%, and 4.4%, respectively, were admitted to the ICU/CCU (**Table 4**). The majority of patients in CTAS categories 1 to 3 were admitted to a hospital ward, while the majority of CTAS 4–5 patients (57.6%) were discharged home from the ED. The proportion of patients discharged directly from the ED was highest in the CTAS 4–5 category and decreased with increasing acuity score to 4.3% in the CTAS 1 group (**Table 4**). Compared to other hospital types, patients with higher acuity scores were more frequently discharged from the ED at teaching hospitals; as many as 7.3% of CTAS 1 patients were discharged directly from the ED at teaching hospitals, while only 3.6% and 4.0% were discharged from the ED in large and small community hospitals, respectively.

### Outcomes Performance of the CTAS

**Figure 1:**
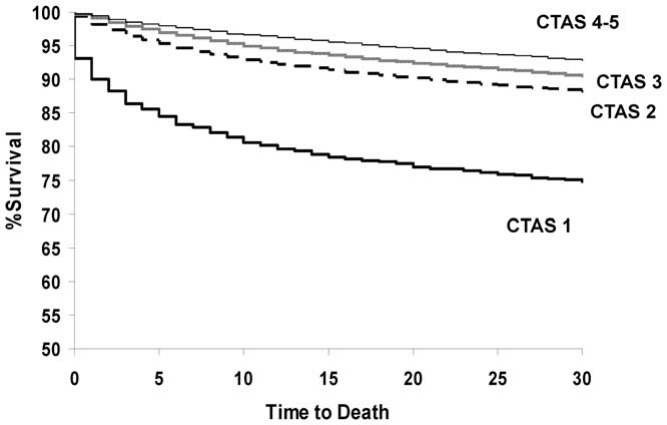
Survival for patients with HF presenting to the ED by CTAS level.

**Figure 2:**
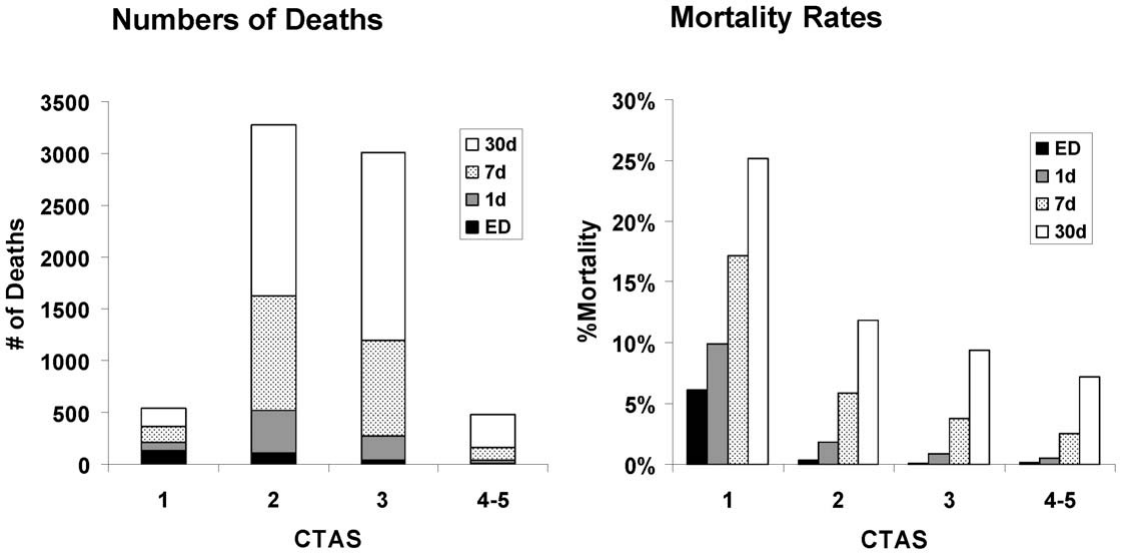
Cumulative number of ED-, 1-day, 7-day, and 30-day deaths (left), and corresponding mortality rates (right) by CTAS level.

CTAS level stratified early mortality among HF patients in the ED (see **Figure 1**). ED death occurred in 6.1% (CTAS 1), 0.4% (CTAS 2), and ≤0.2% (CTAS 3–5) and 1-day mortality was 9.9% (CTAS 1), 1.9% (CTAS 2), 0.9% (CTAS 3), and 0.5% (CTAS 4–5). Overall, 7-day mortality rates were 17.2%, 5.9%, 3.8%, and 2.5%, for CTAS scores 1, 2, 3, and 4–5, respectively. While death rates were higher with greater triage acuity, the absolute number of early deaths was highest in CTAS categories 2 and 3. As shown in **Figure 2**, early deaths accumulated to a greater degree up to 30 days after ED presentation among those in categories 2 and 3, despite the higher mortality rates being observed among those with greater acuity.

**Figure 3:**
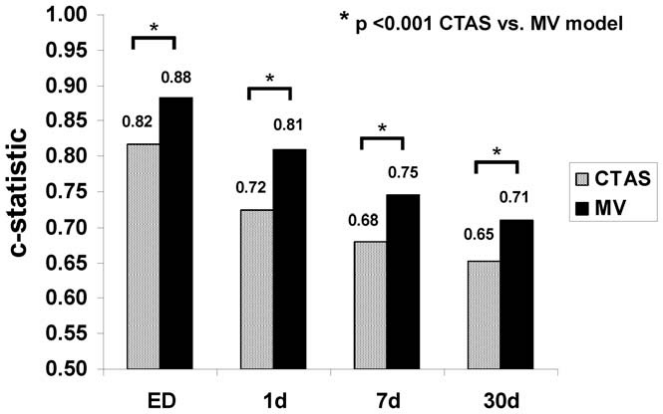
C-statistics for age/sex-adjusted CTAS vs. multivariable model for prediction of ED-, 1-day, 7-day and 30-day death.

Although the age/sex-adjusted CTAS score had high discrimination for emergent death (e.g., 1-day death), performance of the age/sex-adjusted CTAS level decreased with increasing time horizon up to 30 days (**Figure 3**). The performance of the age/sex-adjusted CTAS level was also reduced compared to a previously-published multivariable model [14], which also accounted for cardiac and non-cardiac disease, and ED length of stay (see **Figure 3**, p<0.001 for CTAS vs. multivariable models). There was significant attenuation of the odds ratios for the CTAS and arrival by paramedic in the multivariable models comparing 1-day vs. 7-day death, while other covariates remained stable between early and later time points (see **Table 5**).

**Table 5:** Factors associated with death.

		24-hr Death		7-day Death	
		OR (95% CI)	p-value	OR (95% CI)	p-value
Age	Per 10 years	1.35 (1.26, 1.44)	<0.001	1.45 (1.40, 1.51)	<.001
Male sex		1.30 (1.14, 1.47)	<0.001	1.28 (1.19, 1.38)	<.001
Triage acuity score					
Low acuity	CTAS 4–5	Referent	Referent	Referent	Referent
Urgent	CTAS 3	1.32 (0.93, 1.88)	0.12	1.23 (1.04, 1.45)	0.016
Emergent	CTAS 2	2.41 (1.71, 3.40)	<0.001	1.79 (1.52, 2.11)	<.001
Critical (resuscitation)	CTAS 1	9.06 (6.28, 13.06)	<0.001	4.47 (3.67, 5.45)	<.001
Arrival by paramedic		5.31 (4.43, 6.36)	<0.001	3.30 (3.03, 3.60)	<.001
No. of prior HF admissions:	None	Referent	Referent	Referent	Referent
	1	0.94 (0.77, 1.15)	0.55	0.91 (0.81, 1.03)	0.13
	≥2	0.72 (0.49, 1.05)	0.09	0.84 (0.68, 1.02)	0.08
Valvular heart disease		1.08 (0.85, 1.38)	0.52	1.04 (0.90, 1.19)	0.61
PVD		0.92 (0.70, 1.22)	0.57	0.97 (0.83, 1.13)	0.71
Dementia		1.75 (1.42, 2.16)	<0.001	1.82 (1.61, 2.06)	<.001
Respiratory disease		1.15 (0.98, 1.35)	0.08	1.09 (1.00, 1.20)	0.06
Renal disease		1.04 (0.86, 1.25)	0.73	1.05 (0.94, 1.17)	0.36
Metastatic cancer		3.04 (2.19, 4.23)	<0.001	3.28 (2.67, 4.01)	<.001
ED length of stay	Per 5 hours	0.61 (0.56, 0.67)	<0.001	0.87 (0.84, 0.90)	<.001

### Predictors of Triage-Critical HF

Chart abstraction data were available for a total of 3371 patients. The multivariable associations of triage-critical HF (CTAS category 1), including all model covariates, are shown in the **Table S1**. Factors predicting the highest-acuity score in the parsimonious model included: oxygen saturation <90% with adjusted odds ratio 5.92 (95%CI; 3.09–11.81, p<0.001), New York Heart Association class IV symptoms with odds ratio 5.41 (95%CI; 2.74–11.38, p<0.001), arrival to the ED by paramedic with odds ratio 3.52 (95%CI; 1.70–8.02, p = 0.001), and respiratory rate >24 breaths/minute with odds ratio 1.96 (95%CI; 1.05–3.67, p = 0.034). The c-statistic for the parsimonious model was 0.91 suggesting excellent discrimination, with no lack of model fit (Hosmer and Lemeshow p = 0.692).

## Discussion

In this population-based study, we found that a semi-quantitative disease non-specific triage acuity scale employed in EDs across Canada was able to stratify emergent death in patients with acute HF. The CTAS score had high discrimination for deaths that occurred in the ED or within 1 day of presentation, with greater than 9-fold risk of 1-day mortality in those who were triage critical. Patients who were triage-critical (CTAS 1) demonstrated greater respiratory abnormalities, presented to the ED with paramedic transport, and had worse New York Heart Association class. Surprisingly, a high proportion of triage-critical patients (4.3% overall and 7.3% in teaching hospitals) were discharged directly from the ED; these patients may have initially responded to treatment in the emergency department, and their clinical response may have been interpreted as an indicator of low risk. However, the link between initial response to treatment and improved prognosis has not been demonstrated in patients with HF. Indeed prior work has suggested that there is a substantial overlap in the prognostic profiles of HF patients who are admitted to hospital vs. discharged from the ED [14]. The presence of discharged triage-critical patients, who overall have a high mortality rate, further underscores the need for objective clinical decision tools to guide disposition decisions of HF patients in the ED.

While the CTAS was predictive of emergent outcomes, there were limitations to the scale. Stratification of mortality by CTAS was attenuated in the larger group of non-critical patients, as demonstrated by the small separation in outcomes for patients in CTAS groups 2 to 5 and the higher absolute number of deaths among those with intermediate acuity (e.g., CTAS 2 and 3). Prediction of early mortality at 7 and 30 days was attenuated, potentially reflecting the impact of post-acute care, medical therapy, and health behaviors. While there was a difference in the rates of ED discharge, hospitalization, and ICU admission among the different acuity levels, the scale did not stratify the groups well in this regard, suggesting that decisions regarding a patient's disposition are not usually predicated on CTAS levels. Given the above, there may be limited potential for the CTAS to enable identification of the low-risk patient who is safe for discharge from the ED.

While empirical assessments of prognosis often have limited ability to risk-stratify patients [4], [5], others have shown that nurses' risk ratings may be better than quantitative models [16]. The downstream consequence of inaccurate estimation of outcomes is that there may be a mismatched relationship between care received and perceived patient risk [17], [18]. This is further influenced by patients' expectations, since their perceptions of risk also differ substantially from that of validated models [19]. Statistical risk models enable improved prognosis estimation, and although instruments exist for a number of conditions that are often encountered in the emergency department [20]–[22], there are few tools that have been developed specifically to guide decision-making for patients with acute HF who present emergently.

A validated ED-based HF risk assessment tool has the potential to maximize the allocation of resources for this increasingly prevalent condition and improve patient outcomes. The EFFECT-HF risk model demonstrated the ability to stratify risk at 30-days after hospital admission with an over 10-fold risk in the highest compared to the lowest risk groups [9]. Furthermore, the same model was able to stratify risk over an extended period of follow-up exceeding 5 years [23], and it has been externally validated [10], [24]. Other HF mortality models have examined hospitalized HF patients in whom the decision has already been made to admit the patient to hospital [8], [25], [26], and have not been proven in the wider range of HF patients who present acutely to the ED in whom the disposition has not yet been decided.

The age- and sex- adjusted CTAS score was robust in discriminating death within 1-day of ED presentation, but the prediction of events beyond the emergent phase was attenuated, particularly in the large group of patients who were not triage-critical (CTAS 2–5). The importance of improving mortality prediction for patients within the moderately high acuity groups cannot be overemphasized: while this group (CTAS 2 and 3) had a smaller percentage of early deaths than those who were triage-critical (7-day mortality for CTAS 1 vs. 2–3: 17.2% vs. 4.7%), it had a much larger absolute number of early deaths (367 vs. 2830 at 7 days) than the highest acuity group. A quantitative model that accounts for physiologic and biochemical derangements may improve prognostication for the large group in whom CTAS was not able to adequately stratify early risk. Indeed, predictors of high acuity (CTAS 1) related primarily to respiratory status (dyspnea, tachypnea, and hypoxemia) and mode of transportation to the ED (via ambulance). Features such as blood pressure - routinely obtained during triage - were not associated with CTAS 1, although such parameters have been strongly linked with HF outcomes [27].

The CTAS is applicable for emergent mortality risk estimation in the undifferentiated ED patient with acute HF, and could be used to guide decisions regarding disposition in the ED. For example, patients with the highest acuity score could be admitted to hospital in a monitored setting, given the associated short-term risk of mortality in this group. The primary advantage of CTAS is its simplicity and ease of use, which is a strength in the time-limited setting of initial triage assessment. A practical and rapid risk assessment system is important when timely decisions must be made in the face of competing demands, and an abundance of clinical information may not be available. The CTAS does allow for flexibility according to clinical judgment because the healthcare provider can scale up the acuity level if a patient is perceived to be more unwell than otherwise classified by the scale. However, this may also represent a disadvantage, because the subjectivity involved in such a decision may result in prognosis estimates that are less objective with reduced reproducibility. Interestingly, patients who presented to the ED in triage critical status were less likely to have received care by a physician within the preceding 30 days, which may have influenced the severity of the presentation. This aspect of the transition from community to hospital may be of interest in future studies. Finally, future studies should consider comparison of statistically-derived clinical prediction rules or multivariable regression models against simpler triage acuity scales in acute medical care.

### Study Strengths and Limitations

The strengths of this study included the size of our study cohort, the population-based nature, the broad inclusion criteria, and information about important clinical variables and outcomes. Some limitations of our study should be noted. We could not account for the quality of care provided to patients in the ED which may have modified early outcomes. However, interventions have been shown to have limited impact on acute mortality and thus would likely not have impacted the association of CTAS with emergent outcomes. We focused primarily on symptomatic pulmonary congestion, and isolated systemic congestion without respiratory symptoms was not examined. Furthermore, we did not have access to information about effect modifiers such as health behaviors (e.g., dietary or medication compliance) or the quality of post-discharge care.

In conclusion, we found that the Canadian Triage Acuity Score, a semi-quantitative risk scale, is useful in stratifying the risk of emergent mortality among heart failure patients, occurring up to 1-day following ED presentation. While the scale identifies high-acuity groups of patients who are at high risk of emergent death, it is more limited in stratifying the moderately high to low acuity patient, particularly those who can be safely discharged from the ED. Our study suggests the need for a clinical risk stratification tool that can be used after the initial triage phase to guide decision-making and improve outcomes in HF patients who present to the ED.

## Supporting Information

Table S1 **Nonparsimonious predictors of highest acuity level (CTAS 1 score) in 3371 HF patients with chart abstraction data.**
(DOC)
