# Supplementary material for: Prediction of Emergent Heart Failure Death by Semi-Quantitative Triage Risk Stratification
Source: PLoS One. 2011 Aug 10;6(8):e23065. doi: 10.1371/journal.pone.0023065 (PMC3154275; doi:10.1371/journal.pone.0023065)
Supplement: Table S1 — Nonparsimonious predictors of highest acuity level (CTAS 1 score) in 3371 HF patients with chart abstraction data. (DOC) [file pone.0023065.s001.doc]

**Online Table S1. Nonparsimonious predictors of highest acuity level (CTAS 1 score) in 3371 HF patients with chart abstraction data**

|  | **Odds Ratio (95% CI)** | **p-value** |
| --- | --- | --- |
|  | ORtxt | ProbChiSq |
|  |  |  |
| ***Demographics and presentation*** |  |  |
| Age <70 years | 1.38 (0.61-2.94) | 0.419 |
| Male | 1.28 (0.68-2.46) | 0.444 |
| Transportation via ambulance | 3.47 (1.61-8.18) | 0.002 |
| Presentation to a teaching hospital* | 0.58 (0.25-1.23) | 0.176 |
| Arrival at ED overnight (00:00 to 06:00) | 1.08 (0.48-2.26) | 0.852 |
| ED visit in the past 7 days | 0.33 (0.02-1.81) | 0.318 |
| Hospital discharge in the past 7 days | 1.33 (0.35-3.95) | 0.638 |
| ***Clinical features*** |  |  |
| Systolic blood pressure <100 mmHg | 0.99 (0.21-3.31) | 1.000 |
| Heart rate >120 beats/min | 1.56 (0.63-3.54) | 0.314 |
| Respiratory rate >24 breaths/min | 1.85 (0.96-3.58) | 0.065 |
| Oxygen saturation < 90% | 5.85 (2.98-11.94) | <.001 |
| Respiratory arrest in transit to ED | 20.32 (0.42->99.99) | 0.123 |
| NYHA class IV dyspnea | 4.86 (2.42-10.32) | <.001 |
| Ongoing Chest pain | 1.63 (0.84-3.10) | 0.143 |
| ***Past medical history*** |  |  |
| Shock or respiratory failure | 1.13 (0.15-5.12) | 0.886 |
| Heart failure | 0.42 (0.15-1.04) | 0.075 |
| Myocardial infarction | 1.31 (0.65-2.60) | 0.442 |
| Unstable angina | 0.38 (0.06-1.56) | 0.240 |
| Prior PCI or CABG surgery | 1.05 (0.44-2.37) | 0.910 |
| Aortic or mitral valve disease | 1.21 (0.52-2.58) | 0.642 |
| Hypertension | 0.79 (0.33-1.78) | 0.579 |
| PVD | 3.29 (0.83-10.50) | 0.060 |
| Diabetes | 1.68 (0.87-3.24) | 0.119 |
| Chronic obstructive lung disease / asthma | 1.16 (0.60-2.20) | 0.652 |
| Dementia | 2.52 (0.75-7.21) | 0.103 |
| Rheumatologic disease | 5.23 (0.96-21.51) | 0.033 |
| Liver cirrhosis | 1.51 (0.08-8.25) | 0.700 |
| Renal disease | 1.30 (0.41-3.59) | 0.629 |

* versus large community hospital
